# Case of acute retinal necrosis with rapid progression to proliferative vitreoretinopathy: A case report

**DOI:** 10.1097/MD.0000000000038150

**Published:** 2024-05-17

**Authors:** Shinichiro Chujo, Hisashi Matsubara, Yoshitusgu Matsui, Kumiko Kato, Mineo Kondo

**Affiliations:** a Department of Ophthalmology, Mie University Graduate School of Medicine, Mie, Japan.

**Keywords:** acute retinal necrosis, proliferative vitreoretinopathy, rhegmatogenous retinal detachment, vitrectomy

## Abstract

**Rationale:**

Acute retinal necrosis (ARN) was first reported in 1971 by Urayama et al as an acute uveitis accompanied by retinal arteritis and white retinal lesions in the peripheral retina that can progress to a rhegmatogenous retinal detachment (RRD). We have experienced a case of ARN that, unlike the common developmental course to an RRD associated with ARN, progressed to proliferative vitreoretinopathy (PVR) involving the entire retina in 2 days. The purpose of this report is to present our findings in the case of ARN with an atypical rapid time course.

**Patient concerns:**

The patient was a 56-year-old woman who was treated for uveitis of unknown origin by her primary care physician. She was referred to our hospital because of a worsening of the fundus findings.

**Diagnosis:**

Fundus examination in our hospital revealed vitreous opacities in the right eye, yellowish-white lesions extending around the retina, and some retinal hemorrhages. Because the retinal changes suggested ARN, we performed a polymerase chain reaction of the anterior atrial fluid and detected varicella-zoster virus. Then, the diagnosis of ARN was confirmed, and treatment was begun. At 1 month and a half after beginning the treatment, focal retinal traction was observed in the right fundus. Two days later, a circumferential PVR and a total retinal detachment were detected.

**Interventions:**

We then performed vitrectomy with an encircling buckle and a silicone oil tamponade.

**Outcomes:**

Our examination 6 months postoperatively showed that the retina was attached and the BCVA was 20/200.

**Lessons:**

Our findings of a case of ARN showed that the progression from a local vitreous traction to a full circumferential PVR can develop in 2 days.

## 1. Introduction

Acute retinal necrosis (ARN) was first reported in 1971 by Urayama et al as an acute uveitis accompanied by retinal arteritis and white lesions in the peripheral retina that can progress to a rhegmatogenous retinal detachment (RRD).^[1]^ ARN causes a reduction in vision due to vitreous opacities, vasculitis obliterans, and retinal necrosis in an average of 15 days after the diagnosis.^[2]^

Typically, a vitreous traction-induced retinal tear forms at a necrotic lesion in the periphery of the retina causing an RRD in 1 to 2 months after the diagnosis of ARN.^[3]^ In some cases, complications induced by proliferative vitreoretinopathy (PVR) have also been reported. Thus, Hamid et al reported that an anterior PVR was a common complication in eyes with ARN, and it can affect the treatment outcomes for the RRD.^[4]^ The retinal detachment and its treatment in patients with ARN is important for clinicians because of its significant impact on the visual prognosis of ARN.

We have examined a case of ARN that, unlike the common developmental time of RRD, progressed to PVR involving the entire retina in 2 days. To the best of our knowledge, such a case has not been reported. The purpose of this report is to present our findings in the case of ARN with an atypical rapid time course. We shall present data related to its pathogenesis and management.

## 2. Case report

The patient was a 56-year-old woman who was treated for uveitis of unknown cause by her primary care physician. She was referred to our hospital because of a worsening of the fundus findings.

**Figure 1.**
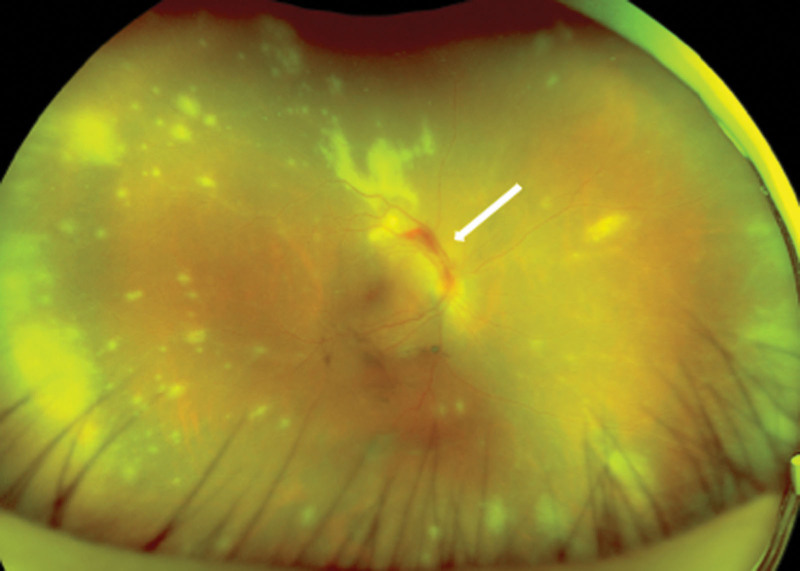
Fundus photograph of a patient diagnosed with ARN. Photograph shows yellowish-white lesions extending around the retina and a hemorrhage (white arrow). ARN = acute retinal necrosis.

Our examination found that her best-corrected visual acuity (BCVA) was 20/100 in the right eye and 20/20 in the left eye. The intraocular pressure was 16 mm Hg in the right eye and 18 mm Hg in the left eye. Slit-lamp examination showed anterior ocular inflammation including keratic precipitates in the right eye. Fundus examination revealed vitreous opacities, yellowish-white lesions extending to the peripheral retina, and some hemorrhages in the retina of the right eye (Fig. 1).

We suspected ARN based on these findings and performed a polymerase chain reaction (PCR) on the aqueous humor. We also began treating the suspected ARN with intravenous acyclovir (30 mg/kg/day), intravenous betamethasone (6 mg/day for 3 days), oral prednisone (30 mg/day), and aspirin (100 mg/day). The PCR of the anterior atrial fluid detected varicella-zoster virus (VZV) to confirm the diagnosis of ARN.

**Figure 2.**
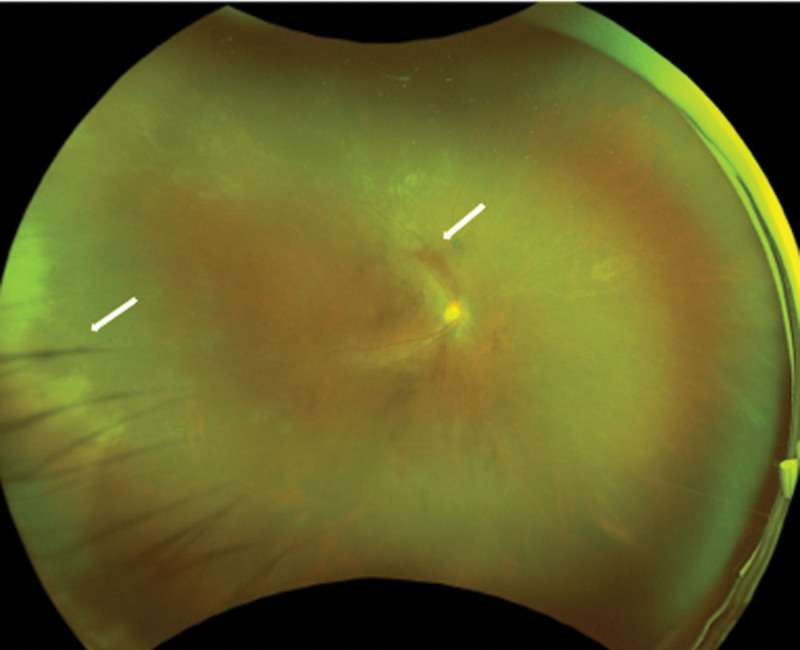
Fundus photograph of a patient diagnosed with ARN. The white retinal lesions have become necrotic. (white arrow). ARN = acute retinal necrosis.

**Figure 3.**
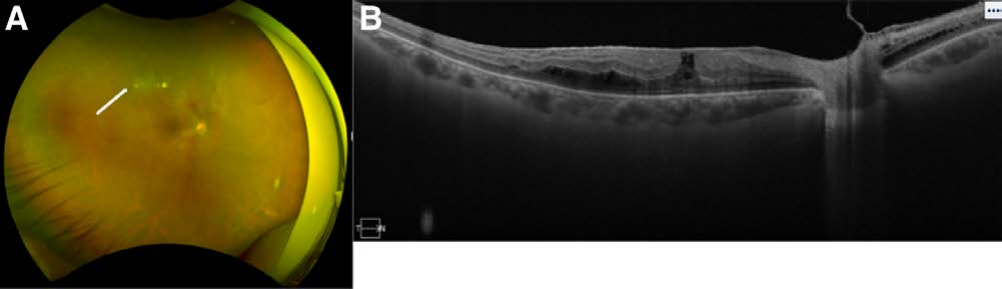
Fundus photograph and OCT image of a patient diagnosed with ARN. (A) Focal retinal traction can be seen (white arrow). (B) OCT image shows that retina was attached. ARN = acute retinal necrosis.

**Figure 4.**
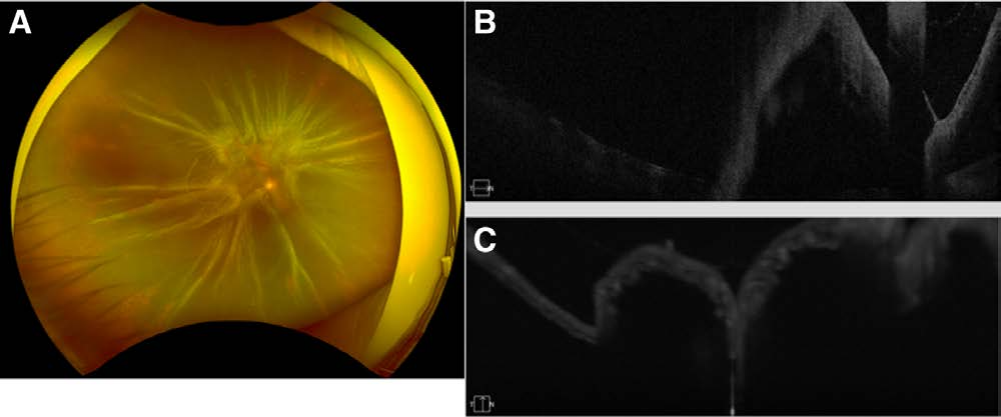
Fundus photograph and OCT image of a patient diagnosed with ARN. (A) Circumferential proliferative vitreoretinopathy and total retinal detachment. (B and C) OCT images show total retinal detachment. ARN = acute retinal necrosis.

One month after the beginning of the treatment, the white retinal lesions became necrotic but there was no retinal detachment. The BCVA improved to 20/40 in the right eye (Fig. 2) At 1 month and a half after the start of the treatments, a focal retinal traction was observed in the right fundus (Fig. 3A and B). We recommended vitrectomy, but the patient refused the surgery. Then the patient was followed with an appointment scheduled 2 days later. At that examination, the fundus had a circumferential PVR and a total retinal detachment (Figs. 4A–C).

**Figure 5.**
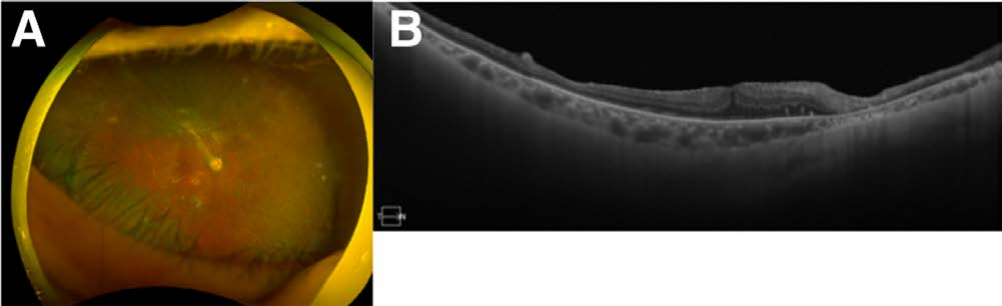
Fundus photograph and OCT image of a patient diagnosed with ARN. (A) Retina is attached after the oil tamponade was removed. (B) OCT image shows that the retina is attached. ARN = acute retinal necrosis.

We then performed a vitrectomy with an encircling buckle and a silicone oil tamponade of the right eye. Our examination 6 months postoperatively showed that the retina was attached and the BCVA was 20/200 (Fig. 5A and B).

## 3. Discussion

We report our findings of a case of ARN that progressed from the ARN to focal vitreous traction of the retina and to a full circumferential PVR. This progression was very rapid and it occurred in 1 month and a half after the diagnosis. The progression to a PVR was expected but the unexpected rapid development was not expected.

In this case, focal retinal traction progressed to PVR in 2 days, and the retina was attached after the surgery. This course is atypical for ARN cases.

The development of a PVR has been reported to be caused by a disruption of the blood-retinal barrier resulting in a migration of retinal pigment epithelial (RPE) cells into the retina and vitreous cavity.^[5]^

Another cause for the PVR was the virulence of VZV because it is known that VZV causes more retinal damage than that caused by herpes simplex virus (HSV).^[6]^

Thus, eyes with ARN associated with VZV are at a high risk of developing PVR because of inflammatory cytokines in the retina due to the retinal damage caused by VZV. Thus, our patient was at a high risk of developing PVR not only because of the RPE cells in the vitreous cavity, but also because of the presence of inflammatory cytokines released due to the severe retinal damage caused by VZV.

The vitreous traction on the retina may have also contributed to the development of the PVR. This may be why the PVR developed so quickly in 2 days.

The question arises on whether prophylactic vitrectomy should have been performed when vitreous traction was first detected. There is currently a lot of discussion on whether prophylactic vitrectomy will reduce the incidence of RRD in eyes with ARN.^[7–10]^ Prophylactic vitrectomy for ARN is considered to be effective because it removes the inflammatory mediators and the vitreous traction on the retina more completely than laser coagulation of the necrotic retina. This combined with tamponading the retina would then prevent the retinal detachment.

Most recently, Shipe et al reported a significant reduction in the incidence of RRD in eyes with ARN following early vitrectomy compared to the eyes who continued to be treated by antiviral medications.^[10]^ On the other hand, other studies have shown that prophylactic vitrectomy for ARN does not significantly reduce the incidence of RRD.^[11,12]^ Of note, Ozdemir et al reported that prophylactic vitrectomy was not helpful in preventing the development of RRD in eyes with ARN.^[12]^

Then, we questioned whether prophylactic vitrectomy should have been performed once the retinal traction was identified. We believed that prophylactic vitrectomy would not have been helpful in our case. The first reason is the location of the necrotic retinal lesions. Ishida et al classified the sites of necrotic lesions in ARN according to Holland classification for cytomegalovirus retinopathy and evaluated whether prophylactic vitrectomy would be effective.^[13]^ The results showed that all patients with lesions in Zone 1 extending from the posterior pole area to the ora serrata developed RRD even after prophylactic vitrectomy.

In our case, the necrotic lesion was located in Zone 1, and the area where the retinal traction occurred was close to Zone 1. Thus, prophylactic vitrectomy would probably not be effective because Zone 1 is the starting point of the disease process.

A second reason why prophylactic vitrectomy would not be helpful in our case is the report of poor postoperative vision caused by the silicon oil tamponade. Sara et al reported that all ARN patients who underwent prophylactic vitrectomy and silicon oil tamponade had poor visual acuity at 6 months postoperatively.^[14]^ Thus, we do not believe that prophylactic vitrectomy would have been beneficial in our case.

## 4. Limitation

The limitation of this report is that surgery was not possible when focal retinal traction was observed. Because the patient did not wish to have surgery at that time.

Further study is needed to determine whether surgery should be performed when focal retinal traction is present.

## 5. Conclusions

We studied a case of ARN that progressed from local vitreous traction to a full circumferential PVR in 2 days. Thus, we recommend that clinicians need to monitor the status of the retina frequently in cases of ARN.

## Acknowledgments

We thank Professor Emeritus Duco I. Hamasaki of the Bascom Palmer Eye Institute of the University of Miami (Miami, FL, USA) for critical discussion and final manuscript revisions.

## Author contributions

**Conceptualization:** Shinichiro Chujo, Hisashi Matsubara, Mineo Kondo.

**Data curation:** Shinichiro Chujo.

**Methodology:** Shinichiro Chujo, Yoshitusgu Matsui.

**Supervision:** Kumiko Kato.

**Validation:** Shinichiro Chujo.

**Writing – original draft:** Shinichiro Chujo.

**Writing – review & editing:** Mineo Kondo.
